# A novel approach for assigning levels to monkey and human lumbosacral spinal cord based on ventral horn morphology

**DOI:** 10.1371/journal.pone.0177243

**Published:** 2017-05-24

**Authors:** Cassandra Gross, Brian Ellison, Aron S. Buchman, Ei Terasawa, Veronique G. VanderHorst

**Affiliations:** 1 Department of Neurology, Division of Movement Disorders, Beth Israel Deaconess Medical Center, Harvard Medical School, Boston, Massachusetts, United States of America; 2 Rush Alzheimer’s Disease Center, Rush University Medical Center, Chicago, Illinois, United States of America; 3 Wisconsin National Primate Research Center, Madison, Wisconsin, United States of America; UniversitatsKlinikum Heidelberg, GERMANY

## Abstract

Proper identification of spinal cord levels is crucial for clinical-pathological and imaging studies in humans, but can be a challenge given technical limitations. We have previously demonstrated in non-primate models that the contours of the spinal ventral horn are determined by the position of motoneuron pools. These positions are preserved within and among individuals and can be used to identify lumbosacral spinal levels. Here we tested the hypothesis that this approach can be extended to identify monkey and human spinal levels. In 7 rhesus monkeys, we retrogradely labeled motoneuron pools that represent rostral, middle and caudal landmarks of the lumbosacral enlargement. We then aligned the lumbosacral enlargements among animals using absolute length, segmental level or a relative scale based upon rostral and caudal landmarks. Inter-animal matching of labeled motoneurons across the lumbosacral enlargement was most precise when using internal landmarks. We then reconstructed 3 human lumbosacral spinal cords, and aligned these based upon homologous internal landmarks. Changes in shape of the ventral horn were consistent among human subjects using this relative scale, despite marked differences in absolute length or age. These data suggest that the relative position of spinal motoneuron pools is conserved across species, including primates. Therefore, in clinical-pathological or imaging studies in humans, one can assign spinal cord levels to even single sections by matching ventral horn shape to standardized series.

## Introduction

The spinal cord is a rostrocaudally heterogeneous structure, which contains diverse inter-related groups of neurons and circuits, each mediating distinct functions. This is obvious for the cervical and lumbosacral enlargements which serve arm and neck control versus leg control. However, even within the enlargements, not all levels serve the same function. For example, the level just rostral to the lumbar enlargement is involved in the initiation of locomotion [1] and distinct levels control flexion, extension or retraction movements during walking [2]. Accurate identification of spinal levels is crucial not only for understanding normal function, but is essential for delineating the location and extent of pathologies which impair function, ranging from spinal cord injury to neurodegenerative diseases such as amyotrophic lateral sclerosis [3], Parkinson’s disease [4, 5], or Alzheimer’s disease [6].

Despite its importance, accurately assigning levels to human spinal cords is complicated by several factors. During autopsy, dorsal root ganglia that can be used to accurately determine spinal level based upon nerve roots (i.e. segmental level) are often not harvested at the lumbosacral level. Even in cases where this is feasible, the organization of spinal segmental levels based upon nerve roots is driven by the anatomy of the lumbosacral plexus and not by the organization of the spinal cord [7]. Depending on the degree of post- or pre-fixation of the lumbosacral plexus, segmental spinal levels may vary by one or more segments. This leads for example to inter-individual differences in the dermatome demarcation [8–10]. In addition, similar to differences in brain weight, there is considerable variability in the length of the spinal cord among human subjects [11, 12]. Finally, complete lumbar enlargement samples are not always available, especially as collection of fresh frozen tissue for genomic and proteomic analysis is becoming standard in brain banking.

In prior work in the cat, we developed an accurate approach for identifying lumbosacral spinal levels that overcomes challenges related to inter-individual differences in size and segmental organization [13]. We first assigned start (0) and end (100) levels based on the position of internal landmarks, one at the rostral and one at the caudal extent of the lumbosacral enlargement. Levels could then be assigned relative to these landmarks and lumbosacral enlargements of individual cases could be accurately aligned. This method was then validated based on the results of retrograde tracing which visualized the spinal locations of motoneuronal cell groups across a large number of animals. In line with earlier studies using chromatolysis [14], motoneuronal cell groups innervating functionally distinct muscle groups formed separate columns, but our work showed that the relative position of these columns appeared fixed between individual animals. Due to this combination of motoneuronal cell groups dictating the shape of the ventral horn and them being organized in a fixed way, it was then possible to recognize the appropriate spinal level based upon the shape of the ventral horn.

It is not known if a similar approach can be employed to assign levels in the primate lumbosacral spinal cord. Work done to date suggests that like other species, human spinal motoneuron groups are strictly organized in longitudinal columns. Based upon cresyl violet staining, 11 columns can be identified, 5 of which are present in the lumbosacral cord [15]. Recent work underscores the importance of recognizing the columnar nature of motoneuron organization within the human spinal cord for localizing postmortem indices of neuropathology [3]. However, data is lacking about whether the organization of motoneuronal cell groups in the primate spinal cord is fixed and whether the ventral horn shape can be used to recognize relative levels and align individual cases.

In the current study, we first visualized iliopsoas and pelvic floor motoneurons in Onuf’s nucleus using retrograde tracing to define rostral and caudal landmarks of the monkey (*Macaca mulatta*) lumbosacral spinal cord. Next, we assessed whether spinal cords with labeled motoneurons best align among cases using a) classical segmental levels which are based on the location of nerve roots that exit the spinal cord, b) spinal cord length based on an absolute distance from the rostral to the caudal end of the lumbosacral enlargement, or c) relative internal landmarks defined by rostral and caudal motoneuron populations that determine ventral horn shape. We then visualized motoneurons in homologous parts of the human (*Homo sapiens*) spinal cord using immunohistochemistry for choline acetyl transferase. Finally, we reconstructed complete human lumbosacral cords using Nissl stains to assess whether the shape of the ventral horn is fixed among cases, and whether spinal levels can be derived using ventral horn shape.

## Material and methods

### Monkey study

We used 7 adult, female rhesus monkeys (average weight 8.3kg; range 6.6–12.8kg, *M*. *mulatta*). All experiments were performed under the guidelines established by the NIH and USDA (NIH Guide for the Care and Use of Laboratory Animals) and were reviewed and approved by the Animal Care and Use Committee, University of Wisconsin, Madison. Animal care was in accordance with the recommendations of the Weatherall report, “The use of non-human primates in research”. All monkeys were used for brainstem-spinal tracing studies as described previously by VanderHorst, et al. [16].

#### Surgical procedures

All surgeries were performed under sterile conditions. Animals were induced with ketamine (0.1mg/kg i.m.), and maintained under isoflurane anesthesia (1.5% i.t.) for the duration of the surgery. They received buprenorphine analgesia prior to and following surgery. After incision of the overlying skin, the iliopsoas and semimembranosus muscles were exposed. Using a Hamilton syringe, we injected 40–50μl 0.5% cholera toxin subunit B (CTb; List Biological Lab,.CA; Cat# 703 in distilled water) into the iliopsoas and semimembranosus muscles. The injection site was then cleaned with sterile saline and the skin was closed. The external anal sphincter was injected with a similar volume without skin incision because the muscle is closely connected to the skin. After 3 days survival time to allow for uptake and transport of the tracer, animals were anesthetized with ketamine (0.1mg/kg; i.m.) and pentobarbital (0.5 mg/kg; i.v.), and transcardially perfused with 2 liters of phosphate-buffered saline (PBS; pH 7.4; 0.1 M; room temperature) followed by 2–3 liters of fixative (2% glutaraldehyde (Sigma—Aldrich, St. Louis, MO; Cat# G5882) and 1% paraformaldehyde (Sigma-Aldrich, St. Louis, MO; Cat# 158127) in 0.1M phosphate buffer; pH 7.4; room temperature). The spinal cords were removed while leaving the dura mater intact, post-fixed for 1–3 hours at room temperature, and stored in PBS (at 4°C).

#### Histology

We cut the spinal cord into segments based upon the organization of the spinal roots leaving the dura mater, with each spinal cut being positioned immediately caudal to the corresponding dorsal rootlets entering the spinal cord. We cut segments T11 to S3 into 5 (M5-7) or 6 (M3-M4, M8-9) series of 80 μm thick transverse sections on a vibratome. One series was used to visualize CTb following a standard immunohistochemcial protocol as described by Vanderhorst et al [16]. Sections were mounted on slides, dehydrated in a graded series of alcohols, and coverslipped with DePeX mounting medium (VWR International, Radnor, PA; Cat# 100503–836).

#### Analysis

Using a Nikon Eclipse Ci microscope (objective 20x; numerical aperture 0.50), we quantified CTb labeled motoneurons defined as labeled cell bodies with one or more dendritic or axonal branches [17]. Because the study’s aim was to determine location rather than precise number, we did not attempt to differentiate among alpha or gamma motoneurons. We then aligned this data using three methods: a) we used an equidistant length down the spinal cord, starting at the rostral end of the L2 segment; b) we aligned the cases at the rostral end of segment L4; c) we used a relative scale based upon a landmark at the rostral end of the enlargement (demarcated by the most rostral CTb labeled iliospoas motoneurons) and one at the caudal end (demarcated by the level where CTb labeled pelvic floor motoneurons in Onuf’s nucleus touch the ventral horn). Following analysis, of the 7 Monkeys M3-M9, we omitted M3 and M4 due to spurious labeling of motoneurons following the hamstring injections.

To visualize the gradual change in morphology of the spinal ventral horn, we compiled schematic drawings by tracing the boundaries of the gray matter of the right half side of every third section in a single 1:5 series throughout the entire rostrocaudal extent of the lumbosacral enlargement. We used a Zeiss microscope with camera lucida under darkfield illumination (objective 3.2x/numerical aperture 0.50). We digitized these drawings into Adobe Illustrator (CC2015) using a Wacom tablet (Intuos 4XL PTK-1240). Finally, we plotted labeled iliopsoas motoneurons and pelvic floor motoneurons (case M5; each drawing containing the information of 3, serial 1:5 sections) into this series, to indicate the exact location of the rostral (iliopsoas) and caudal (Onuf’s nucleus) landmarks.

### Human study

We obtained 3 human formalin fixed spinal cords from autopsy cases at Beth Israel Deaconess Medical Center, Boston. This study was considered exempt as determined by the Institutional Review Board of BIDMC. Subjects included a 62 year old woman with biphenotypic leukemia who died from sepsis, a 63 year old man with a history of alcohol abuse who died from sepsis in the setting of an aspiration pneumonia, and an 87 year old woman with a clinical and pathological diagnosis of Dementia with Lewy Bodies who died from cardiac arrest. This latter case, which contains alpha-synuclein pathology in the lumbosacral cord ([18] case number 8), was included to assess whether the morphology of the ventral horn is preserved sufficiently in older subjects with neurodegenerative disease affecting the spinal cord.

We cut the lumbosacral spinal cords on a freezing microtome into 12 series of 80 μm thick sections. We prepared one series for immunohistochemistry by heating the tissue to 90 degrees Celsius in 10% Tris-buffered saline (pH10) for 30 minutes. After cooling for 1 hour, the tissue was treated with 1% sodium borohydride in phosphate buffered saline (PBS; 0.1M, pH7.4) for 30 minutes to remove excessive aldehydes, followed by 1% H_2_O_2_ in PBS for 30 minutes. Tissue was incubated in 10% nonfat dry milk (NFDM) in PBS with 0.3% Triton-X (PBT) for 2 hours before being transferred to a solution of 2% NFDM in PBS with 0.3% PBT, containing the primary polyclonal antibody choline acetyl transferase (ChAT 1:100 host Gt; EMD Millipore, Billerica, MA; Cat# AB144P). Tissue was incubated in primary antibody for 3 nights at room temperature before being transferred to a solution containing biotinylated donkey anti-goat secondary antibody (Affinipure Donkey anti-goat IgG 1:500 polyclonal; Jackson Immuno, West Grove, PA; Cat# 705065147) in PBT-azide for 1 night. Tissue was then incubated in Avidin/Biotin Complex (Vectastain elite ABC kit 1:500; Vector Laboratories Inc, Burlingame, CA; Cat# PK6100) in PBT for 2 hours. Reaction product was visualized with 0.04% 3,3-Diaminobenzidine tetrahydrochloride hydrate (DAB; Sigma Aldrich, St. Louis, MO; Cat# D5637), 0.2% Nickel Sulfate Hexahydrate in sterile water (Sigma Aldrich, St. Louis, MO; Cat# N4882) in 0.003% H_2_O_2_ and PBS for 15 min. We used this series to determine whether rostral and caudal landmarks as defined in the monkey study matched with ChAT-IR clusters of neurons in the human spinal cord.

To visualize the gradual change in morphology of the spinal ventral gray, we counter-stained 2 series with Thionin [19]. Drawings and reconstructions of the spinal gray matter were made similar to the monkey reconstructions. We then aligned each of the three spinal cord series based on the anatomical landmarks at levels 0 and 100. Cryoprotection, which was only applied in the human study, resulted in 10% shrinkage as measured in 6 intact lumbar segments (2 segments per case, in 3 cases) prior and after cryoprotection in sucrose solution. Shrinkage related to histological processing from moment of tissue cutting (80μm) to slide preparation resulted in an additional 44% shrinkage in both monkey and human tissues (35μm +/-3μm; 3 sections per case, in 5 monkeys; 3 sections per case, in 3 human cases). However, as section thickness prior to cutting was used to reconstruct the length of the lumbar enlargement, these processing changes did not affect final estimations. The effects of tissue processing were kept consistent within human and monkey studies due to the use of strict protocols.

## Results

### Identification of rostral and caudal lumbosacral landmarks in monkey and human spinal cord

**Fig 1:**
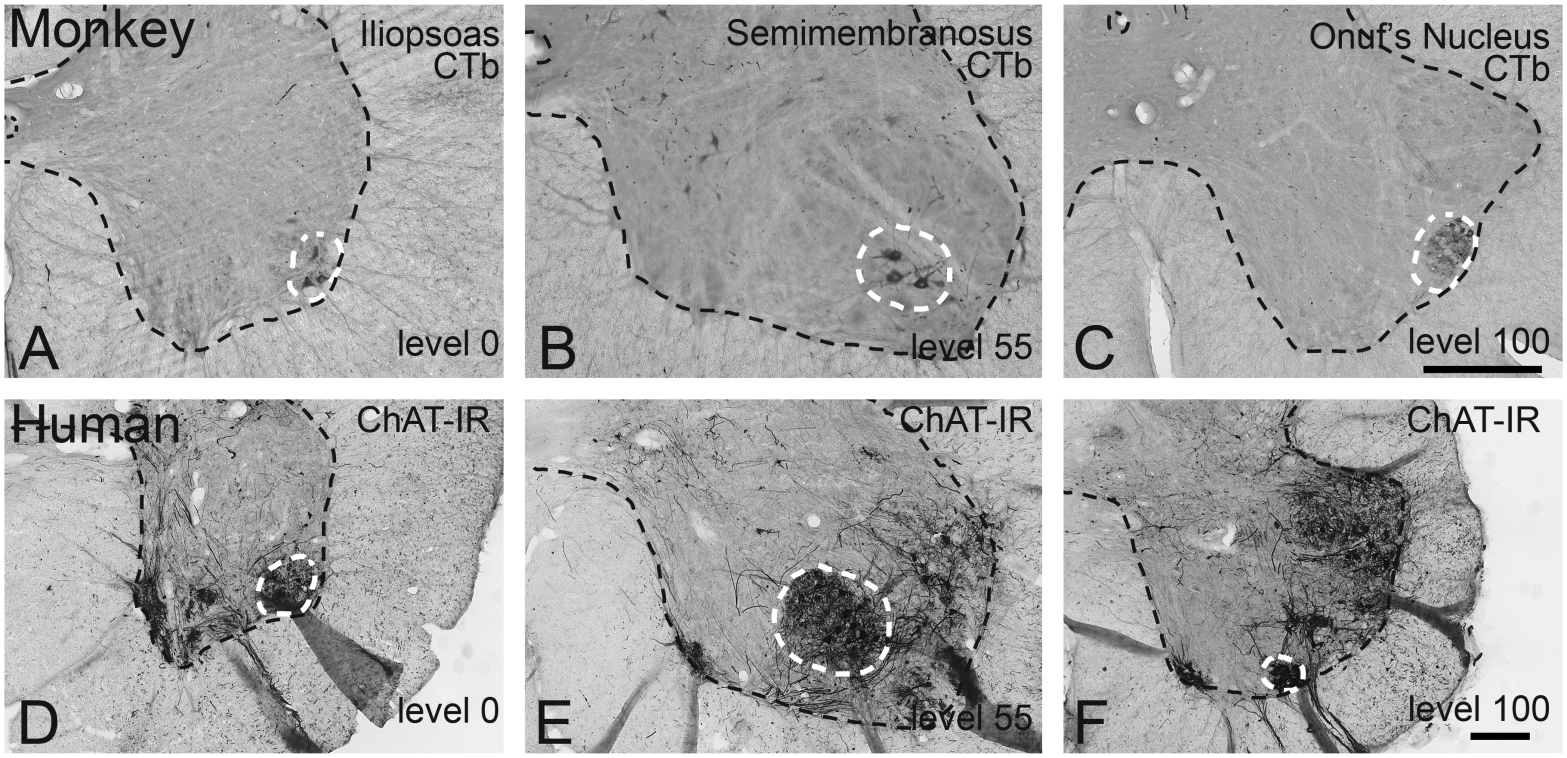
Identification of rostral and caudal landmarks in the lumbosacral enlargement in rhesus monkey and humans. (A-C) Retrogradely labeled motoneurons in the rhesus monkey lumbosacral cord following CTb injections into the (A) iliopsoas, (B) semimembranosus, and (C) external sphincter muscle. Note that the iliopsoas motoneuron pool demarcates the rostral end of the enlargement, where the ventral horn extends laterally. The caudal landmark is demarcated by pelvic floor motoneurons of the external sphincter (Onuf’s nucleus) touching the edge of the ventral gray matter. (D-F) ChAT-IR neurons in the human lumbosacral cord (87 year old woman with Dementia with Lewy Bodies). Panels (D-F) are homologous to panels (A-C) in the monkey. Note that the changes in shape of the ventral horn, demarcating the rostral and caudal landmarks, can be identified in the human spinal cord. Similar to monkey, these changes in shape are determined by (ChAT-IR) motoneurons, similar to the rhesus monkey. Bars in (A-C), and (D-F) = 500μm.

In monkeys, retrogradely (CTb) labeled motoneurons innervating the iliopsoas muscle formed the most rostral group of motoneurons that defines the lateral portion of the ventral horn. The presence of these motoneurons caused the ventral horn to bulge out in its ventrolateral corner (Fig 1A). This level demarcates level 0. Retrogradely labeled motoneurons innervating the semimembranosus formed a cluster in the center of the ventral horn at mid lumbar levels (Fig 1B). Retrogradely labeled motoneurons innervating the external anal sphincter were found in Onuf’s nucleus. This group was located in the center of the ventral horn at its most rostral level, but as the ventral horn diminished in size ventrolaterally, it was located at the border between gray and white matter (Fig 1C). This level demarcates level 100.

In human spinal cords, we identified a homologous rostral landmark where the ventral horn protrudes ventrolaterally solely based upon morphology. This protrusion indeed contained the most rostral group of ChAT-IR neurons that defines the lateral part of the ventral horn (Fig 1D). This level demarcates level 0. Caudally, Onuf’s nucleus can be identified based upon its relatively small sized neurons either via Nissl or ChAT stain. In unstained tissue, Onuf’s nucleus appears hyperdense under darkfield illumination compared to surrounding tissue. The level where it contacts the ventro-lateral border between the gray and white matter defines level 100 (Fig 1F). The presumed hamstring/semimembranosus motoneuronal cell group was apparent at mid lumbar levels based on ChAT-IR motoneurons clustered in the center of the ventral horn (Fig 1E).

### Alignment of the monkey spinal cord based upon length, segmental level or internal landmarks

We then assessed which method serves best to align monkey lumbosacral cords across different cases. We used 3 approaches based upon absolute length, classical segmental levels, or internal landmarks, and compared the alignment of rostrocaudal distributions of retrogradely labeled iliopsoas, semimembranosus and external anal sphincter motoneurons among cases.

**Fig 2:**
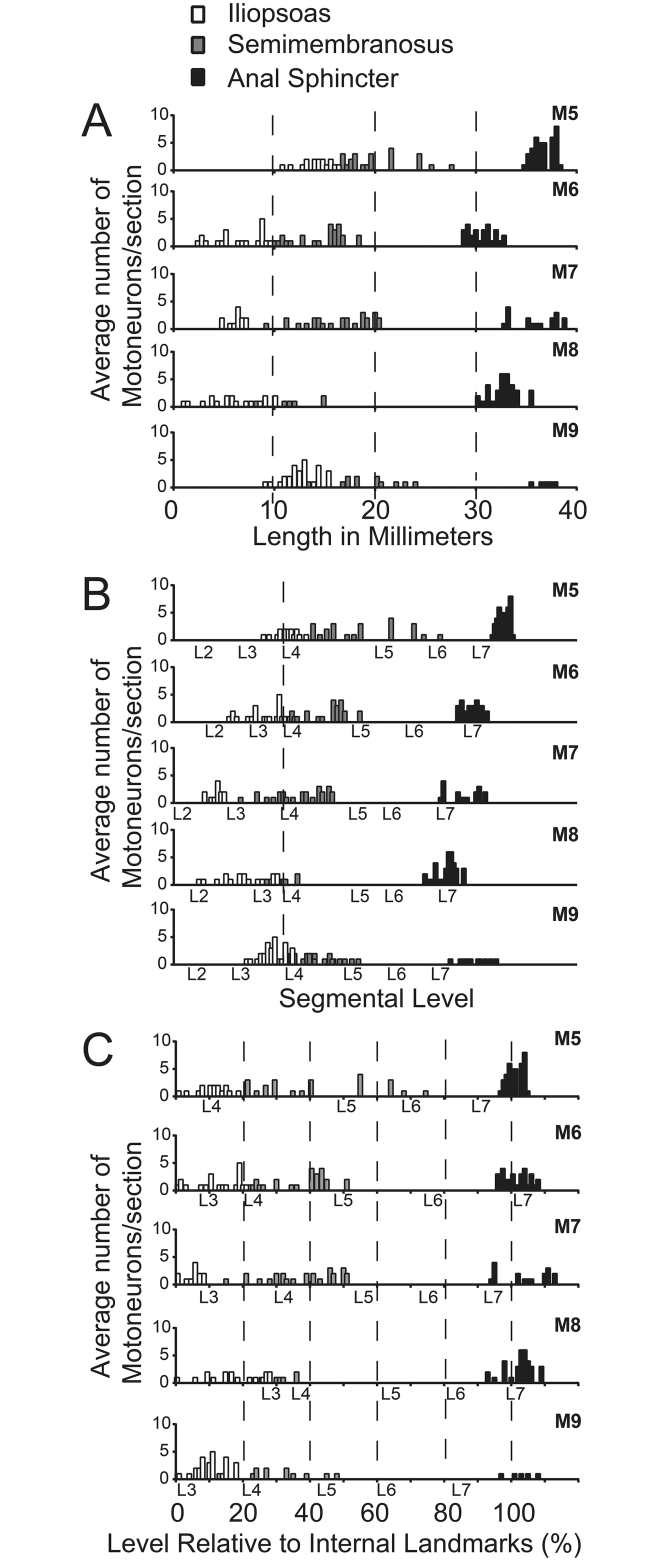
Comparison of three approaches to align the distribution of non-human primate lumbosacral motoneurons. Rostrocaudal distribution of iliopsoas, semimembranosus, and external sphincter motoneurons in 5 monkeys. Spinal cords were aligned among cases based upon (A) absolute length (millimeter distance calculated from the thickness of each section and the number of sections); (B) segmental level, starting at L4, and (C) internal landmarks representing level 0 (defined by the ventrolateral edge of the ventral horn extending laterally and representing the presence the most rostral leg motoneurons) and level 100 (defined by the ventral gray border changing from a curved protrusion to a straight edge or Onuf’s nucleus touching the ventral white matter. Note the size differences in (A) and the segmental differences in (B), which contrast the similarity in distribution of motoneurons across cases in (C).

Alignment of the lumbosacral enlargement based upon absolute length (one rostral anchor at L2) resulted in mismatches of rostrocaudal motoneuron distributions among cases. At the rostral end, labeled iliopsoas motoneuron populations were located more rostrally in cases M6-8 compared to M5 and M9 (Fig 2A). Caudally in the enlargement, the external sphincter group in M6 was present rostral to M8. In M5, M7, and M9, it was present caudal to M8.

The segmental organization also differed markedly among monkeys, and resulted in shifts of up to a full segmental level among cases (Fig 2B). The rostral end of the iliopsoas motoneuron group was present rostrally in the L2 segment (M8), caudally in L2 (M6 and M7), rostrally in L3 (M5 and M9). Semimembranosus motoneurons were present at the transition between L3 and L4 (M7 and M8), L4 (M6 and M9), or the transition between L4 and L5 (M5). Motoneurons in Onuf’s nucleus were located in L6 (M6 and M8), at the transition between L6 and L7 (M7), or in L7 (M5 and M9).

Alignment using a relative 0–100 scale, based upon the rostral pole of the iliopsoas motoneuron group (level 0) and the external sphincter motoneuron group touching the gray-white border (level 100) resulted in matched distributions of all three motoneurons groups (iliopsoas, semimembranosus, external sphincter) across all five cases (Fig 2C).

These results show that a relative subdivision of the lumbosacral enlargement based upon internal landmarks serves best to align non-human primate spinal cords among different cases.

### Changes in ventral horn shape rostrocaudally along the lumbosacral enlargement across species

**Fig 3:**
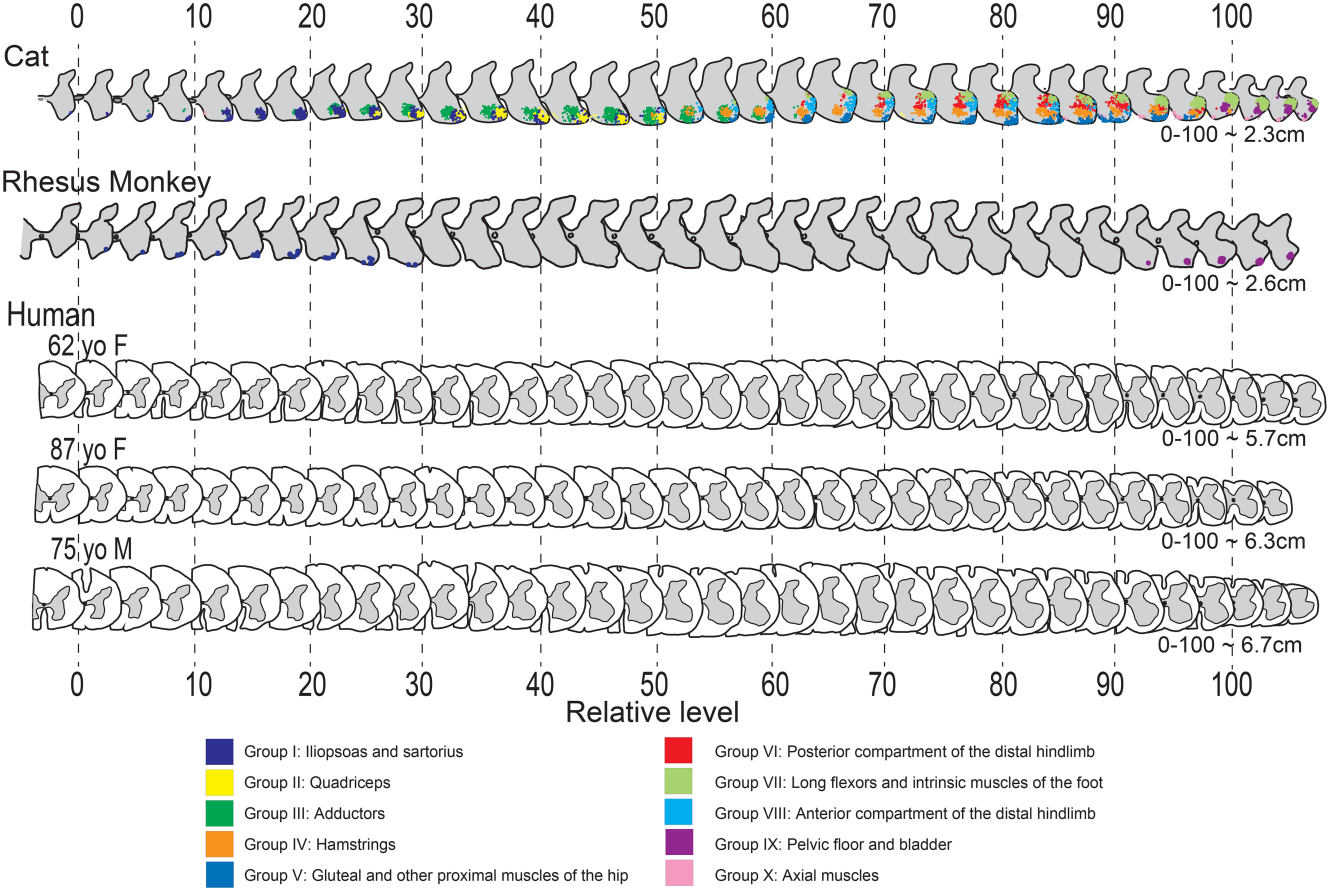
Schematic drawings of lumbosacral enlargement sections in cat, monkey and human aligned via internal landmarks. Each schematic drawing represents the right side of every third section in a 1:5 series of 80μm sections (M5) or in a 1:6 80μm series in all 3 human cases. Cat data, with motoneuronal cell groups serving functionally distinct muscles marked by distinct colors, was adapted with permission from Vanderhorst and Holstege [13]. In all 3 species, series were aligned according to rostral (level 0) and caudal (level 100) anatomical landmarks as defined in Fig 1. Lengths signify distances calculated in centimeters from level 0 to 100, based upon the thickness of each section and the number of sections, and after corrections for tissue shrinkage. Note that consistent changes in ventral horn shape are congruent across all 3 species, and among the 3 human subjects, despite obvious differences in size.

We then addressed the questions whether internal landmarks can be identified in spinal cords in which tracing is not feasible and whether a relative scale serves to align such spinal cords. In 3 human spinal cords, we obtained a series of schematic drawings of the shape of the gray matter through the entire lumbosacral enlargement from sections that were spaced at regular intervals. We then identified the rostral and caudal landmarks, based upon the morphology of the ventral horn as outlined above, representing level 0 and level 100, respectively (Fig 3). Using this approach, the gradual changes in shape of the ventral horn among the 3 human subjects matched well, despite differences in length and size of the lumbosacral cord, age and gender of the subjects, or presence of neurodegenerative disease (Dementia with Lewy Bodies with involvement of the spinal cord; see case 8 from [see case 8 from18]). In addition, the gradual changes in shape of the ventral horn along the rostrocaudal extent of the lumbosacral cord were remarkably consistent among species (Fig 3), including human, monkey and cat [13]. Of note, these shapes are not exactly the same across species, reflecting species differences. For example, the medial ventral horn at caudal levels in the human spinal cord (85–100) is not as well developed as compared to cat and monkey (Figs 1 and 3). This is most likely due to the absence of motoneurons innervating tail muscles in human.

## Discussion

Assigning rostrocaudal levels to the human lumbosacral enlargement that does justice to the internal, functional organization has been challenging, given marked variations in segmental levels and size of the enlargement among subjects. In this study we tested whether an alternative approach that had been previously developed and validated in the cat [13] can be applied to the human post mortem spinal cord without additional complex staining protocols.

We show that the shape of the ventral horn in the human lumbosacral enlargement can be used as a guide to approximate rostrocaudal levels among different cases, irrespective of length, size and segmental level. This approach is based upon the notion that a) motoneurons determine the shape of the ventral horn in the lumbosacral enlargement, b) the position of motoneuronal cell groups in the lumbosacral enlargement is fixed relative to each other, c) internal landmarks can be identified without markers to serve as rostral and caudal anchors to subdivide the enlargement into relative levels. We will discuss this in more detail below, in addition to strengths, limitations, and potential applications.

### Subdividing the lumbosacral enlargement into relative levels

The approach we present takes advantage of the preserved organization of lumbosacral motoneuron pools among species, including in cat [13, 14], rat [20], mouse [21], monkey and human (current study). The iliospoas motoneuronal cell group was chosen as the rostral landmark in both cat [13] and monkey (this study) as this cell group represents the most rostral cluster of hindlimb motoneurons in the lumbar enlargement. Though studies involving the position of iliopsoas motoneurons in other species are sparse, psoas motoneurons also consistently form a compact cluster in the ventrolateral corner of the ventral horn of the rostral part of the enlargement in rat and mice [20, 21]. Species differences relate largely to the assigned segmental level, further supporting the advantage of using a relative scale to compare levels rather than segmental levels. As for the choice of the caudal landmark, Onuf’s nucleus stands out in the caudal enlargement as it consists of densely packed pelvic floor motoneurons that are smaller than motoneurons innervating leg muscles (in human: [22, 23, 24], this study; non-human primate: [25, 26], this study; and cat: [13]). The level 100 landmark, i.e. Onuf’s nucleus touching the ventrolateral border of the ventral horn, can therefore be reliably recognized with a standard counterstain or with darkfield illumination in unstained tissues. In other species including rodents, motoneurons innervating the urethral sphincter and ischiocavernosus muscle are located in a similar position forming the dorsolateral nucleus [27], and this nucleus may therefore be used as a landmark in these species. Similar to variation in segmental levels of psoas motoneurons, segmental levels of pelvic floor motoneurons vary as described in both monkey and human [24, 26].

Motoneuronal cell groups themselves dictate the shape of the ventral horn as is best illustrated by comprehensive mapping in the cat [13] as shown in Fig 3. The ventral horn shape then becomes a useful guide as the organization of motoneuronal cell groups is fixed relative to each other [13], and independent of absolute length or of the segmental level which is based on anatomical landmarks external to the CNS. We now show that this fixed organization is not only present in non-primate species, but also in the monkey. As human spinal cord can be well aligned based upon relative changes in ventral horn morphology, it is likely that a fixed organization also holds for the human spinal cord.

To define a relative scale to subdivide the spinal cord into levels, as an alternative to segmental levels, it is important to identify landmarks that are consistent among individual cases and preferably also among species. As we show in this manuscript, the suitability as well as the consistent and universal nature of the chosen rostral (iliopsoas) and caudal (Onuf’s) landmarks visualized in ventral horn morphology facilitates cross species comparisons and translational studies. These landmarks define a relative scale that can be used to find homologous levels despite individual and species variability. This is especially relevant as there are obvious size differences across species and as the segmental distribution across species varies due to differences in number of thoracic, lumbar or sacral vertebral levels. As a result of the latter differences, the enlargements start and end at different segmental levels across these species (i.e. human L1, monkey L2, cat L3-4), even though the internal organization of the enlargement itself appears more congruous.

### Strengths and limitations

The approach is robust in that it circumvents differences in spinal cord length, size, and variability in segmental levels based on traditional anatomical landmarks external to spinal cord. Furthermore, the approach to assigning spinal levels based on the morphology of the ventral horn can be employed across species, and can be used to assign a level to a single section by comparing it to a standardized series. For example, using the reconstructed human series to first estimate the relative level, motoneuron mapping in cat can then guide the presence of distinct motoneuron groups at that level, in the absence of direct, experimental approaches to identify motoneuron subtypes in humans. In addition, this approach is unburdened from relying on absolute measures which make analysis prone to complications resulting from varied processing protocols. This is because the organization of motoneurons within the spinal cord, and thus the position of internal landmarks using a relative scale, is not affected by the method of processing or visualization, whether this includes free floating histological processing, paraffin embedded tissue analysis, or in vivo or post mortem magnetic resonance imaging.

A limitation is that changes in shape of the ventral horn occur gradually and together with differences in samples among cases it is therefore necessary to observe a range of +/-10 accuracy for assigning relative spinal levels (0–100). Another limitation is that we have no direct means to identify human motoneurons in terms of muscles they innervate given that tracing experiments are not feasible. Therefore we cannot provide direct evidence of the organization of motoneuron groups being fixed similar to cat and monkey. Lastly, aging [28], and neurodegenerative diseases such as Parkinson’s disease and Dementia with Lewy Bodies [4, 18], Alzheimer’s Disease [6, 29], and Multiple System Atrophy [30] affect the spinal cord. Based upon sparse quantitative data, loss of motoneurons is mild [28, 29]. Indeed in the case with Dementia with Lewy Bodies (panels D-F in Fig 1), there were sufficient numbers of lumbosacral motoneurons as visualized with a ChAT stain to allow identification of landmarks despite the abundance of phosphorylated alpha-synuclein (see also case 8 in [18]). However, in cases with motoneuron disease leading to loss of limb motoneurons [31], or diseases which can be accompanied by loss of Onuf’s motoneurons (multiple system atrophy [32]; Machado-Joseph disease [33]), this method may not be feasible.

### Potential applications

Potential applications for this approach include clinical-pathological studies where specific clinical observations are correlated with pathology related to distinct spinal levels. This could for example include correlations between ventral horn size or motoneuron numbers at a particular spinal level, and muscle pathology or muscle strength tested in a distinct muscle ante-mortem. As pointed out above, the approach can be valuable for translational studies, as it allows alignment of levels based upon functional organization rather than segment name. In addition, this approach may aid clinical research. The resolution of current magnetic resonance imaging (MRI) is sufficient to recognize the shape of the ventral horn [34], which likely is a better indicator for functional-anatomical level than vertebral level as is the standard in radiology.

Altogether, this novel approach can serve as a clinically relevant and experimentally useful tool for assigning accurate levels to the human lumbosacral spinal cord and allows for comparison of specific spinal cord levels across individuals and species.
